# Atrial fibrillation mimicking ventricular fibrillation confuses an automated external defibrillator

**DOI:** 10.1007/s12471-018-1098-0

**Published:** 2018-03-12

**Authors:** M. Hulleman, M. T. Blom, A. Bardai, H. L. Tan, R. W. Koster

**Affiliations:** 1 Department of Cardiology, Academic Medical Center—Heart Center, Amsterdam, The Netherlands; 2 Department of Clinical Genetics, Academic Medical Center, Amsterdam, The Netherlands

**Fig. 1:**
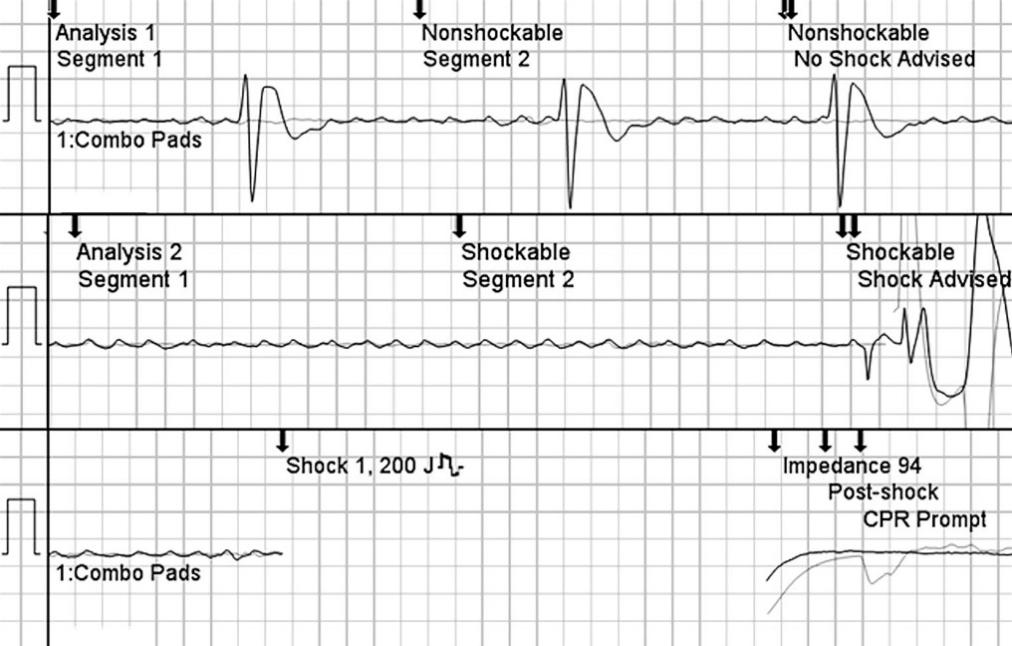
Electrocardiogram of automated external defibrillator showing initial rhythm assessment (panel 1), and second rhythm check after two minutes (panel 2) resulting in a shock (panel 3)

A 47-year-old man suffered an out-of-hospital cardiac arrest. For scientific purposes, we analysed the electrocardiogram of the deployed automated external defibrillator (AED) [1]. Initially, the AED detected a non-shockable rhythm, caused by atrial fibrillation (AF) with high-degree atrioventricular block and slow ventricular escape rhythm (Fig. 1 panel A). During continued rhythm analysis, however, no escape beats occurred and a shock was delivered because isolated AF waves were erroneously interpreted as ventricular fibrillation (VF) (Fig. 1 panels B–C). In-hospital AF mimicking VF in monitored patients has been described before [2, 3], but ours is the first report of inappropriate AED therapy for AF mimicking VF during out-of-hospital cardiac arrest. The sensitivity and specificity of AED algorithms is related to the defibrillation threshold as defined in the AED algorithm [4]. The defibrillation threshold in the AED in this case report was 0.08 mV, which is lower than other AEDs where it ranges from 0.1 to 0.2 mV. An amplitude below the threshold is considered asystole. In this case the AF waves were between 0.08 and 0.2 mV, interpreted as ‘fine’ VF.

It is important to recognise that in current clinical practice we do not routinely retrieve and analyse AED electrocardiograms, and AED shocks are generally used as a proxy for VF. This practice may result in treatment errors. Clearly, AED electrocardiograms should always be analysed to allow for correct clinical decision-making.
